# Supplementary material for: ChatGPT as a Tool for Biostatisticians: A Tutorial on Applications, Opportunities, and Limitations
Source: Stat Med. 2025 Oct 23;44(23-24):e70263. doi: 10.1002/sim.70263 (PMC12548020; doi:10.1002/sim.70263)
Supplement: Supplementary file 1 — Data S1. Supporting Information. [file SIM-44-0-s001.zip › Supplementary Materials Additional.zip!/3.2_Diagnostic_Test/3.2_Diagnostic_Chat9.pdf]

Estimating Sensitivity and Specificity

I would like to determine sensitivity and specificity of two diagnostic tests. I do have the diagnostic test outcomes, but I do not know the gold standard. What can I do?

Great and important question — this is a classic problem in diagnostic test evaluation when no gold standard is available. Without a gold standard, you cannot directly compute sensitivity and specificity in the traditional way (which relies on comparing test results to the true disease status).

But there are **alternative methods** that can help you *estimate* the sensitivity and specificity:

1️⃣ Latent Class Models (LCMs)

- LCMs treat the true disease status as a *latent (unobserved) variable*.
- You model the joint distribution of the observed test outcomes as a function of unknown sensitivities, specificities, and the (unknown) disease prevalence.
- In the simplest case (two tests, one population), you can use a **2-test 1-population LCM** to estimate:
    - Sensitivity and specificity of each test
    - Disease prevalence

Assumptions:

- The two tests are *conditionally independent* given the true disease status.
    - If this is not plausible, you need more complex models (e.g. adding a dependence structure).

Example model structure:

```text
Pr(Test1=+, Test2=+ | disease present) = sensitivity1 × sensitivity2
Pr(Test1=-, Test2=- | disease absent) = specificity1 × specificity2
... etc for other combinations
```

You can fit this model using:

- **Frequentist approaches** (e.g. `poLCA` package in R)
- **Bayesian approaches** (e.g. `rjags`, `Stan`), which are often more flexible

---

2️⃣ Multiple Populations (if available)

If you can collect data from **two or more populations** that are believed to have different disease prevalences:

- You can fit **multi-population latent class models** (Hui-Walter models), which are more robust to test dependence.
- This design helps to relax the conditional independence assumption.

3️⃣ Bayesian Prior Information

If you have **prior knowledge** (from literature or expert opinion) about:

- Prevalence
- Sensitivity and specificity of one of the tests
  You can incorporate this in a Bayesian LCM and obtain more stable estimates.

4️⃣ Partial Gold Standard / Composite Reference Standard

Sometimes people build a **composite reference standard**:

- e.g. patient is considered diseased if either test is positive and clinical signs are present
- This can be useful, but you need to account for *imperfect reference* in your estimation.

Summary: what can you do?

Option	Requirements	Tools
**Latent Class Model (LCM)**	Outcomes of ≥2 tests	R, JAGS, Stan
**Hui-Walter Model (multi-population)**	≥2 populations with different prevalence	R, JAGS, Stan
**Bayesian LCM with priors**	Prior information	R, JAGS, Stan
**Composite Reference**	Clinical knowledge	Custom modeling

If you tell me a bit more about your situation:

✅ How many tests do you have?
✅ How many subjects?
✅ Do you have 1 or more populations?
✅ Any prior knowledge about prevalence / test accuracy?

... then I can suggest the **best model** and even provide **concrete R code** for you! Would you like to continue? 🚀

Please, tell me more about the latent class analysis.

Of course — let's go deeper into **Latent Class Analysis (LCA)** (or more specifically, Latent Class Models for diagnostic test evaluation). It's a very elegant approach for exactly your situation.

🌟 Key Idea of Latent Class Models

The *true disease status* is not observed → it is treated as a **latent (unobserved) categorical variable** (usually 0 = no disease, 1 = disease).

You do observe the **results of one or more diagnostic tests** for each individual.

You model the **joint distribution of the observed test results**, assuming they depend probabilistically on the latent disease status.

The model estimates:

- **Prevalence** of the disease
- **Sensitivity** and **specificity** of each test

🏗️ Basic Model Structure

Example: Two tests (Test 1 and Test 2), one population

For each individual i, let:

- $D_i \in \{0, 1\}$ = latent disease status
- $T1_i \in \{0, 1\}$ = result of Test 1
- $T2_i \in \{0, 1\}$ = result of Test 2

Model parameters:

- $\pi = P(D = 1)$ = prevalence of disease
- Se_1, Se_2 = sensitivity of Test 1 and Test 2
- Sp_1, Sp_2 = specificity of Test 1 and Test 2

Conditional probabilities:

If $D = 1$ (disease present):

- $P(T1 = 1|D = 1) = Se_1$
- $P(T2 = 1|D = 1) = Se_2$

If $D = 0$ (disease absent):

- $P(T1 = 1|D = 0) = 1 - Sp_1$
- $P(T2 = 1|D = 0) = 1 - Sp_2$

📊 Observed Data

You typically have counts of each test combination:

Test 1	Test 2	Count
+	+	n_{11}
+	-	n_{10}
-	+	n_{01}
-	-	n_{00}

🔢 Model Likelihood

Each combination's probability is:

$$P(T1, T2) = P(D = 1) \cdot P(T1, T2|D = 1) + P(D = 0) \cdot P(T1, T2|D = 0)$$

Assuming **conditional independence** given disease status:

$$P(T1, T2|D) = P(T1|D) \cdot P(T2|D)$$

🔎 Estimation

You can fit this model:

- **Maximum Likelihood** → `poLCA` in R
- **Bayesian** (with prior distributions) → `rjags`, `rstan`

The model returns **posterior estimates** (Bayesian) or **MLEs** for:

- π (prevalence)
- Se_1, Se_2
- Sp_1, Sp_2

⚠️ Important Assumptions

1️⃣ **Conditional independence** of tests given the disease status

- If violated, need extended models (e.g. with test dependence terms).

2️⃣ **Model identifiability**

- For 2 tests in 1 population → model is just-identifiable but sensitive.
- Better if ≥2 populations with different prevalence → Hui-Walter model → more robust.

🔧 Basic R Example (2 tests, using `poLCA`)

```R
# Install package
install.packages("poLCA")
library(poLCA)
```

```
# Example: Test result combinations
# Variables must be coded as factors
data <- data.frame(
  T1 = factor(c(1,1,0,0)),  # 1 = positive, 0 = negative
  T2 = factor(c(1,0,1,0)),
  count = c(50, 30, 20, 100)  # example counts
)

# Latent class model with 2 classes (disease status 0/1)
f <- cbind(T1, T2) ~ 1

# Fit latent class model
model <- poLCA(f, data, nclass = 2, freq = data$count)

# Print results
print(model)
```

This will estimate:

- Prevalence
- Se and Sp for each test

📚 When to use more complex models

Situation	Recommended Model
2 tests, 1 population	Simple LCM (but fragile)
≥2 populations with different prevalence	Hui-Walter LCM
Dependent tests	LCM with dependence (e.g. random effects or direct dependence terms)
Prior knowledge available	Bayesian LCM

🚀 Summary

- **Latent Class Models** let you estimate sensitivity, specificity, and prevalence *without a gold standard*.
- Very useful when gold standard is unknown or imperfect.
- Requires modeling assumptions (conditional independence is key).
- Best used with ≥2 populations or multiple tests to ensure identifiability and robustness.

If you tell me more about your data:

✅ **How many tests** do you have?
✅ **How many subjects**?
✅ **One or multiple populations**?
✅ Would you prefer **frequentist** or **Bayesian** approach?

I can **write you a tailored example in R** to get you started! Want me to? 🚀

I have two populations with two different disease prevalences. I have two diagnostic tests which are conditional independent. I would like to get the log-likelihood of this setting.

Perfect — you are describing the **Hui-Walter latent class model** — the classical setting for estimating Se/Sp without gold standard when you have **two populations with different prevalences**, two conditionally independent tests, and binary test outcomes.

Let's write down the model carefully — and then I will give you the **full formula for the log-likelihood**.

✨ Model Setup

You have:

✅ Two populations → $j = 1, 2$

✅ Two diagnostic tests → $k = 1, 2$

✅ Condition: **Tests are conditionally independent given disease status**

Parameters to estimate:

For each **population** j:

- π_j = prevalence in population j

For each **test** k:

- Se_k = sensitivity of test k
- Sp_k = specificity of test k

Observed data:

For each population j, you observe counts of all 4 test outcome combinations:

Test 1	Test 2	Population j	Count n_{jk}
+	+	j	$n_{j,11}$
+	-	j	$n_{j,10}$
-	+	j	$n_{j,01}$
-	-	j	$n_{j,00}$

📐 Model Probabilities

For each population j, the probability of each combination of test outcomes is:

$$P(T_1 = t_1, T_2 = t_2 \mid \text{pop} = j) = \pi_j \cdot P(T_1 = t_1 \mid D = 1) \cdot P(T_2 = t_2 \mid D = 1) + (1 - \pi_j) \cdot P(T_1 =$$

Let's define for convenience:

- $q_1(t_1) = Se_1^{t_1}(1 - Se_1)^{1-t_1}$
- $q_2(t_2) = Se_2^{t_2}(1 - Se_2)^{1-t_2}$
- $r_1(t_1) = (1 - Sp_1)^{t_1} Sp_1^{1-t_1}$
- $r_2(t_2) = (1 - Sp_2)^{t_2} Sp_2^{1-t_2}$

Then:

$$P_j(t_1, t_2) = \pi_j \cdot q_1(t_1) \cdot q_2(t_2) + (1 - \pi_j) \cdot r_1(t_1) \cdot r_2(t_2)$$

🧮 Full Log-Likelihood

Now, the full **log-likelihood** is the sum over both populations and all test combinations:

$$\log L = \sum_{j=1}^{2} \sum_{t_1=0}^{1} \sum_{t_2=0}^{1} n_{j,t_1 t_2} \cdot \log \left[P_j(t_1, t_2)\right]$$

🔢 Explicit form

Putting everything together:

$$\log L = \sum_{j=1}^{2} \Bigg[n_{j,11} \cdot \log\left(\pi_j \cdot Se_1 \cdot Se_2 + (1 - \pi_j) \cdot (1 - Sp_1) \cdot (1 - Sp_2)\right) +$$

$$n_{j,10} \cdot \log\left(\pi_j \cdot Se_1 \cdot (1 - Se_2) + (1 - \pi_j) \cdot (1 - Sp_1) \cdot Sp_2\right) +$$

$$n_{j,01} \cdot \log\left(\pi_j \cdot (1 - Se_1) \cdot Se_2 + (1 - \pi_j) \cdot Sp_1 \cdot (1 - Sp_2)\right) +$$

$$n_{j,00} \cdot \log\left(\pi_j \cdot (1 - Se_1) \cdot (1 - Se_2) + (1 - \pi_j) \cdot Sp_1 \cdot Sp_2\right) \Bigg]$$

🚀 Summary

- You are modeling **8 cells** in total (4 test outcomes × 2 populations)
- You estimate **6 parameters**:
  - π_1, π_2
  - Se_1, Se_2
  - Sp_1, Sp_2

If you'd like, I can also:

1️⃣ Write this out as a **vectorized R function** (easy to maximize with `optim`)
2️⃣ Show you how to fit it **in Stan** (Bayesian)
3️⃣ Show you how to fit it **with `poLCA`** (frequentist)

👉 Just tell me which option you'd like to see! Want me to write you the R code for this log-likelihood? 🚀

What is the Hui-Walter paradigm?

Excellent — let's go through it carefully.

🌟 The Hui-Walter Paradigm

The **Hui-Walter paradigm** (Hui & Walter, *Biometrics*, 1980) is a foundational method for estimating **sensitivity and specificity of diagnostic tests** when there is **no gold standard** available.

It is a special case of a **latent class model**.

⚙️ Basic setup

✅ **Two (or more) populations** with *different disease prevalences*
✅ **Two (or more) diagnostic tests**
✅ Tests are assumed to be **conditionally independent** given the true (latent) disease status
✅ True disease status is **unobserved** (latent variable)

🔍 What does it do?

It allows you to **estimate sensitivity and specificity** of each test and disease prevalence in each population — **without needing a gold standard**.

🏗️ Why it works (identifiability)

- If you use **one population and one test**, or even **two tests in one population**, the model is often **not identifiable** — too many unknowns.
- But if you have:
  - **2 populations** with **different prevalence**
  - **2 tests** that are conditionally independent
    then the model becomes **just-identified** → the parameters can be estimated!

📊 Parameters

For each population $j = 1, 2$:

- π_j = prevalence in population j

For each test $k = 1, 2$:

- Se_k = sensitivity of test k
- Sp_k = specificity of test k

🧮 Observed data

For each population → **counts of 4 possible test outcomes**:

Test 1	Test 2	Count n_{j,t_1t_2}
+	+	$n_{j,11}$
+	-	$n_{j,10}$
-	+	$n_{j,01}$
-	-	$n_{j,00}$

📐 Model probabilities

In population j, probability of each test outcome (t_1, t_2) is:

$$P_j(t_1, t_2) = \pi_j \cdot P(t_1, t_2 \mid D = 1) + (1 - \pi_j) \cdot P(t_1, t_2 \mid D = 0)$$

Assuming **conditional independence**:

$$P(t_1, t_2 \mid D) = P(t_1 \mid D) \cdot P(t_2 \mid D)$$

🚀 Why is this useful?

Key insight of Hui & Walter:
Having **two populations with different prevalence** gives **extra information** → makes the model **identifiable**.

Without this, the model with two tests in one population cannot fully separate prevalence from test accuracy.

🧠 How many parameters and equations?

Parameters to estimate:

Parameter	Number
π_1, π_2 (prevalences)	2
Se_1, Se_2 (sensitivities)	2
Sp_1, Sp_2 (specificities)	2
**Total**	**6 parameters**

Observed data:

- For **each population**, you observe **4 outcome probabilities** → 3 degrees of freedom (since probabilities sum to 1).
- Two populations → **6 degrees of freedom** → exactly enough to estimate **6 parameters**.

Result: model is just-identified!

📚 Summary

Concept	Content
**Hui-Walter paradigm**	Use ≥2 populations with different prevalence to estimate Se, Sp, and prevalence without gold standard
**Assumptions**	Conditional independence of tests given true disease status
**Strength**	Allows identifiability with only 2 tests
**Classic reference**	Hui & Walter (1980), *Biometrics*

✨ When is it used?

✅ When no perfect gold standard exists
✅ When tests are cheap to apply
✅ When you can sample different populations (e.g. high-risk vs low-risk)

If you'd like, I can also show:

1️⃣ Example of a Hui-Walter **identifiability proof**
2️⃣ Example **simulation** (how it works in practice)
3️⃣ Example **R code** to fit it (MLE or Bayesian)

👉 Would you like me to give an example or some code? Just tell me! 🚀

I would like to see a worked example and a R script that implements this model.

Perfect — let me give you a **fully worked example** of the Hui-Walter model and a **clear R script** that implements it step by step.

🎓 Scenario (worked example)

You have **two populations**:

- Population 1 → high prevalence
- Population 2 → low prevalence

You apply **two tests** to all subjects.

You observe the following counts:

Population	Test 1	Test 2	Count
Pop 1	+	+	50
Pop 1	+	-	20
Pop 1	-	+	15
Pop 1	-	-	100
Pop 2	+	+	30
Pop 2	+	-	10
Pop 2	-	+	25
Pop 2	-	-	200

🧮 Goal

Estimate:

- π_1, π_2 (prevalence in pop 1 and 2)
- Se_1, Se_2 (sensitivity of Test 1 and 2)
- Sp_1, Sp_2 (specificity of Test 1 and 2)

🖥️ R script to implement Hui-Walter model

I will show you how to:

✅ Write the **log-likelihood function**
✅ Use **optim()** to find MLE estimates
✅ Print results

Here is the full R script:

```r
# Hui-Walter example in R

# Observed counts
counts <- list(
  pop1 = c(n11 = 50, n10 = 20, n01 = 15, n00 = 100),
  pop2 = c(n11 = 30, n10 = 10, n01 = 25, n00 = 200)
)

# Log-likelihood function
loglik <- function(par, counts) {
  # Parameters
  pi1 <- par[1]
  pi2 <- par[2]
  Se1 <- par[3]
  Sp1 <- par[4]
  Se2 <- par[5]
  Sp2 <- par[6]

  # Probabilities for each test outcome in each population
  # Population 1
  p11_1 <- pi1 * Se1 * Se2 + (1 - pi1) * (1 - Sp1) * (1 - Sp2)
  p10_1 <- pi1 * Se1 * (1 - Se2) + (1 - pi1) * (1 - Sp1) * Sp2
  p01_1 <- pi1 * (1 - Se1) * Se2 + (1 - pi1) * Sp1 * (1 - Sp2)
  p00_1 <- pi1 * (1 - Se1) * (1 - Se2) + (1 - pi1) * Sp1 * Sp2
```

```
  # Population 2
  p11_2 <- pi2 * Se1 * Se2 + (1 - pi2) * (1 - Sp1) * (1 - Sp2)
  p10_2 <- pi2 * Se1 * (1 - Se2) + (1 - pi2) * (1 - Sp1) * Sp2
  p01_2 <- pi2 * (1 - Se1) * Se2 + (1 - pi2) * Sp1 * (1 - Sp2)
  p00_2 <- pi2 * (1 - Se1) * (1 - Se2) + (1 - pi2) * Sp1 * Sp2

  # Add small constant to avoid log(0)
  eps <- 1e-10

  # Log-likelihood
  ll <- 0
  ll <- ll + counts$pop1["n11"] * log(p11_1 + eps)
  ll <- ll + counts$pop1["n10"] * log(p10_1 + eps)
  ll <- ll + counts$pop1["n01"] * log(p01_1 + eps)
  ll <- ll + counts$pop1["n00"] * log(p00_1 + eps)

  ll <- ll + counts$pop2["n11"] * log(p11_2 + eps)
  ll <- ll + counts$pop2["n10"] * log(p10_2 + eps)
  ll <- ll + counts$pop2["n01"] * log(p01_2 + eps)
  ll <- ll + counts$pop2["n00"] * log(p00_2 + eps)

  return(-ll)  # because optim does minimization
}

# Initial parameter guesses
# (pi1, pi2, Se1, Sp1, Se2, Sp2)
init <- c(0.5, 0.1, 0.8, 0.9, 0.8, 0.9)

# Bounds for parameters (optional, can also use unconstrained optim)
lower <- c(0, 0, 0, 0, 0, 0)
upper <- c(1, 1, 1, 1, 1, 1)

# Optimize
fit <- optim(
  par = init,
  fn = loglik,
  counts = counts,
  method = "L-BFGS-B",
  lower = lower,
  upper = upper,
  control = list(trace = 1, maxit = 1000)
)

# Print results
cat("Estimated parameters:\n")
cat("pi1 =", fit$par[1], "\n")
cat("pi2 =", fit$par[2], "\n")
cat("Se1 =", fit$par[3], "\n")
cat("Sp1 =", fit$par[4], "\n")
cat("Se2 =", fit$par[5], "\n")
cat("Sp2 =", fit$par[6], "\n")
```

🚀 How to run it

1️⃣ Copy this code into an R script or RStudio
2️⃣ Run it — it will estimate the 6 parameters using **maximum likelihood**
3️⃣ The output will print the **estimated Se, Sp, and prevalences**

📝 Summary

- This is a **direct implementation of the Hui-Walter model**
- You can **easily adapt it** to:
  - More populations

- More tests
- Add dependence terms (if needed)
- Switch to **Bayesian** version (with Stan or JAGS)

Would you like me to also provide:

✅ A **Bayesian version** (Stan / rstan)?
✅ A version with **confidence intervals** (bootstrap)?
✅ A **simulation example** to test the model?

Just tell me! 🚀
It's a great example — very practical for your use case.

I would like to add confidence intervals to the estimated sensitivities, specificities, and prevalences.

Great! Adding **confidence intervals (CIs)** is an important next step — it gives you a sense of the **precision** of your estimates.

Since we used `optim()` to compute **maximum likelihood estimates (MLEs)**, there are two classic ways to get CIs:

🚀 Option 1: Wald-type CIs using the Fisher Information Matrix

- The Hessian (second derivative matrix) at the MLE approximates the **inverse of the Fisher information matrix**.
- The **variance-covariance matrix** of the MLEs is:

$$\text{Var}(\hat{\theta}) \approx [\text{Hessian}]^{-1}$$

- Then approximate **95% CI** for each parameter is:

$$\hat{\theta} \pm 1.96 \times \text{SE}(\hat{\theta})$$

🚀 Option 2: Parametric Bootstrap

- Simulate new datasets from the fitted model
- Re-fit model to each simulated dataset
- Use empirical percentiles of bootstrap estimates for CIs

Advantage: much more robust if the likelihood is not perfectly quadratic (which is common in latent class models).

⚙️ I will show you both options!

🔵 Option 1: Wald-type CIs

Just add this code **after the `optim()` call**:

```r
# Compute Hessian numerically
library(numDeriv)

# Get Hessian
hess <- hessian(func = loglik, x = fit$par, counts = counts)

# Invert Hessian to get variance-covariance matrix
vcov_mat <- solve(hess)

# Standard errors
se <- sqrt(diag(vcov_mat))

# 95% Wald confidence intervals
lower_CI <- fit$par - 1.96 * se
upper_CI <- fit$par + 1.96 * se

# Print results with CIs
param_names <- c("pi1", "pi2", "Se1", "Sp1", "Se2", "Sp2")
cat("Parameter estimates with 95% Wald CIs:\n")
for (i in 1:6) {
  cat(param_names[i], ": ", fit$par[i],
      " (", lower_CI[i], ", ", upper_CI[i], ")\n")
}
```

🔵 Option 2: Parametric Bootstrap

Here's a simple **bootstrap loop**:

```r
# Number of bootstrap samples
n_boot <- 1000

# Store bootstrap estimates
boot_estimates <- matrix(NA, nrow = n_boot, ncol = 6)

set.seed(123)  # for reproducibility

for (b in 1:n_boot) {
  # Simulate new counts from the fitted model

  # First get probabilities from MLE estimates
  par <- fit$par

  pi1 <- par[1]
  pi2 <- par[2]
  Se1 <- par[3]
  Sp1 <- par[4]
  Se2 <- par[5]
  Sp2 <- par[6]

  # Probabilities of each cell
  probs_pop1 <- c(
    pi1 * Se1 * Se2 + (1 - pi1) * (1 - Sp1) * (1 - Sp2),
    pi1 * Se1 * (1 - Se2) + (1 - pi1) * (1 - Sp1) * Sp2,
    pi1 * (1 - Se1) * Se2 + (1 - pi1) * Sp1 * (1 - Sp2),
    pi1 * (1 - Se1) * (1 - Se2) + (1 - pi1) * Sp1 * Sp2
  )

  probs_pop2 <- c(
```

```
      pi2 * Se1 * Se2 + (1 - pi2) * (1 - Sp1) * (1 - Sp2),
      pi2 * Se1 * (1 - Se2) + (1 - pi2) * (1 - Sp1) * Sp2,
      pi2 * (1 - Se1) * Se2 + (1 - pi2) * Sp1 * (1 - Sp2),
      pi2 * (1 - Se1) * (1 - Se2) + (1 - pi2) * Sp1 * Sp2
    )

    # Simulate new counts (multinomial)
    n_pop1 <- sum(counts$pop1)
    n_pop2 <- sum(counts$pop2)

    new_counts_pop1 <- rmultinom(1, size = n_pop1, prob = probs_pop1)
    new_counts_pop2 <- rmultinom(1, size = n_pop2, prob = probs_pop2)

    new_counts <- list(
      pop1 = c(n11 = new_counts_pop1[1], n10 = new_counts_pop1[2], n01 = new_counts_pop1[3], n00 =
  new_counts_pop1[4]),
      pop2 = c(n11 = new_counts_pop2[1], n10 = new_counts_pop2[2], n01 = new_counts_pop2[3], n00 =
  new_counts_pop2[4])
    )

    # Re-fit model to bootstrap sample
    boot_fit <- optim(
      par = init,
      fn = loglik,
      counts = new_counts,
      method = "L-BFGS-B",
      lower = lower,
      upper = upper,
      control = list(maxit = 1000)
    )

    # Store bootstrap estimates
    boot_estimates[b, ] <- boot_fit$par
  }

  # Compute 95% bootstrap percentile CIs
  boot_CIs <- apply(boot_estimates, 2, quantile, probs = c(0.025, 0.975))

  # Print bootstrap CIs
  cat("Bootstrap 95% CIs:\n")
  for (i in 1:6) {
    cat(param_names[i], ": ", fit$par[i],
        " (", boot_CIs[1,i], ", ", boot_CIs[2,i], ")\n")
  }
```

📚 Summary of Options

Method	Pros	Cons
Wald (Hessian)	Fast, simple	Approximate, can be inaccurate if likelihood is not quadratic
Bootstrap	Robust, more accurate	Slower (need to run many optimizations)

🚀 Final recommendation

Since **latent class models can be tricky**, I strongly recommend using **bootstrap CIs** if you can afford the compute time.

Would you like me to also:

✅ Show a **full combined R script** with everything?
✅ Show how to do the **Bayesian version** with proper credible intervals (in Stan)?

Just tell me — happy to help! 🚀

I would like to reparametrize my model using a logistic parametrization for the prevalences, sensitivities, and specificities.

Excellent idea — **logistic reparametrization** is very common and elegant:

✅ It **automatically enforces the constraints**:

- $\pi_j, Se_k, Sp_k \in (0,1)$
- No need for bounds in `optim()`
- The model is **smooth** and often easier to optimize

✅ It is also ideal if you later want to extend to a **Bayesian model** (priors on logit scale are easier to define).

🚀 How to do it

You reparametrize:

$$\pi_j = \text{expit}(\theta_{\pi_j}) = \frac{1}{1+e^{-\theta_{\pi_j}}}$$

$$Se_k = \text{expit}(\theta_{Se_k}) = \frac{1}{1+e^{-\theta_{Se_k}}}$$

$$Sp_k = \text{expit}(\theta_{Sp_k}) = \frac{1}{1+e^{-\theta_{Sp_k}}}$$

🏗️ Updated model

Now, instead of optimizing:

$$(\pi_1, \pi_2, Se_1, Sp_1, Se_2, Sp_2)$$

you optimize:

$$(\theta_{\pi_1}, \theta_{\pi_2}, \theta_{Se_1}, \theta_{Sp_1}, \theta_{Se_2}, \theta_{Sp_2})$$

and **transform** these back inside the likelihood.

🖥️ R code (logistic parametrization)

I will show you how to update the `loglik()` function.

```r
# Logistic (expit) function
expit <- function(x) {
  1 / (1 + exp(-x))
}
```

```r
# Log-likelihood function with logistic parametrization
loglik_logit <- function(par, counts) {
  # Transform parameters
  pi1 <- expit(par[1])
  pi2 <- expit(par[2])
  Se1 <- expit(par[3])
  Sp1 <- expit(par[4])
  Se2 <- expit(par[5])
  Sp2 <- expit(par[6])

  # Probabilities for each test outcome in each population
  # Population 1
  p11_1 <- pi1 * Se1 * Se2 + (1 - pi1) * (1 - Sp1) * (1 - Sp2)
  p10_1 <- pi1 * Se1 * (1 - Se2) + (1 - pi1) * (1 - Sp1) * Sp2
  p01_1 <- pi1 * (1 - Se1) * Se2 + (1 - pi1) * Sp1 * (1 - Sp2)
  p00_1 <- pi1 * (1 - Se1) * (1 - Se2) + (1 - pi1) * Sp1 * Sp2

  # Population 2
  p11_2 <- pi2 * Se1 * Se2 + (1 - pi2) * (1 - Sp1) * (1 - Sp2)
  p10_2 <- pi2 * Se1 * (1 - Se2) + (1 - pi2) * (1 - Sp1) * Sp2
  p01_2 <- pi2 * (1 - Se1) * Se2 + (1 - pi2) * Sp1 * (1 - Sp2)
  p00_2 <- pi2 * (1 - Se1) * (1 - Se2) + (1 - pi2) * Sp1 * Sp2

  # Add small constant to avoid log(0)
  eps <- 1e-10

  # Log-likelihood
  ll <- 0
  ll <- ll + counts$pop1["n11"] * log(p11_1 + eps)
  ll <- ll + counts$pop1["n10"] * log(p10_1 + eps)
  ll <- ll + counts$pop1["n01"] * log(p01_1 + eps)
  ll <- ll + counts$pop1["n00"] * log(p00_1 + eps)

  ll <- ll + counts$pop2["n11"] * log(p11_2 + eps)
  ll <- ll + counts$pop2["n10"] * log(p10_2 + eps)
  ll <- ll + counts$pop2["n01"] * log(p01_2 + eps)
  ll <- ll + counts$pop2["n00"] * log(p00_2 + eps)

  return(-ll)  # because optim does minimization
}
```

🚀 How to fit

Now you simply run:

```r
r


# Initial guesses on logit scale
# logit(0.5) = 0
# logit(0.8) = log(0.8 / 0.2) ≈ 1.386
# logit(0.9) = log(0.9 / 0.1) ≈ 2.197

init_logit <- c(0, 0, 1.386, 2.197, 1.386, 2.197)

# No need for bounds!
fit_logit <- optim(
  par = init_logit,
  fn = loglik_logit,
  counts = counts,
  method = "BFGS",  # unconstrained now
  control = list(trace = 1, maxit = 1000)
)

# Transform back to probability scale
pi1_est <- expit(fit_logit$par[1])
pi2_est <- expit(fit_logit$par[2])
Se1_est <- expit(fit_logit$par[3])
Sp1_est <- expit(fit_logit$par[4])
Se2_est <- expit(fit_logit$par[5])
```

```r
Sp2_est <- expit(fit_logit$par[6])

# Print estimates
cat("Estimated parameters (probability scale):\n")
cat("pi1 =", pi1_est, "\n")
cat("pi2 =", pi2_est, "\n")
cat("Se1 =", Se1_est, "\n")
cat("Sp1 =", Sp1_est, "\n")
cat("Se2 =", Se2_est, "\n")
cat("Sp2 =", Sp2_est, "\n")
```

🚀 How to get CIs (Wald)

Same idea — but now the **Hessian is on the logit scale** — so after inversion:

```r
r

# Hessian
hess_logit <- hessian(func = loglik_logit, x = fit_logit$par, counts = counts)

# Invert Hessian
vcov_logit <- solve(hess_logit)

# Standard errors on logit scale
se_logit <- sqrt(diag(vcov_logit))

# Wald CI on logit scale → transform to probability scale
logit_CI_lower <- fit_logit$par - 1.96 * se_logit
logit_CI_upper <- fit_logit$par + 1.96 * se_logit

# Transform to probability scale
expit_CI_lower <- expit(logit_CI_lower)
expit_CI_upper <- expit(logit_CI_upper)

# Print results
cat("Wald CIs (probability scale):\n")
param_names <- c("pi1", "pi2", "Se1", "Sp1", "Se2", "Sp2")
for (i in 1:6) {
  cat(param_names[i], ": ", expit(fit_logit$par[i]),
      " (", expit_CI_lower[i], ", ", expit_CI_upper[i], ")\n")
}
```

📝 Summary

✅ **Logistic parametrization**:

- Makes optimization much smoother
- Enforces parameter constraints
- Prepares model for Bayesian analysis

🚀 Next steps

If you'd like, I can also show:

✅ How to do a **bootstrap** in this parametrization
✅ How to implement the **Bayesian version** in Stan (with logistic parametrization — very elegant and standard)
✅ How to **simulate data** for testing this model

Would you like me to generate a **full final combined R script** for you with:
✅ logistic model
✅ MLE
✅ Wald CIs
✅ Bootstrap option

🚀 It will be very handy and complete — ready to use! Just tell me. 🚀

Please update the bootstrap function too.

Of course — very good point: when using the **logistic parametrization**, the bootstrap function needs to match this parametrization.

Here's the updated **bootstrap loop** for your logistic model:

🚀 Bootstrap with logistic parametrization

```r
# Number of bootstrap samples
n_boot <- 1000

# Store bootstrap estimates (on probability scale)
boot_estimates <- matrix(NA, nrow = n_boot, ncol = 6)

set.seed(123)  # for reproducibility

for (b in 1:n_boot) {
  # Simulate new counts from the fitted model

  # Use current best estimates (on probability scale)
  pi1 <- expit(fit_logit$par[1])
  pi2 <- expit(fit_logit$par[2])
  Se1 <- expit(fit_logit$par[3])
  Sp1 <- expit(fit_logit$par[4])
  Se2 <- expit(fit_logit$par[5])
  Sp2 <- expit(fit_logit$par[6])

  # Probabilities of each cell
  probs_pop1 <- c(
    pi1 * Se1 * Se2 + (1 - pi1) * (1 - Sp1) * (1 - Sp2),
    pi1 * Se1 * (1 - Se2) + (1 - pi1) * (1 - Sp1) * Sp2,
    pi1 * (1 - Se1) * Se2 + (1 - pi1) * Sp1 * (1 - Sp2),
    pi1 * (1 - Se1) * (1 - Se2) + (1 - pi1) * Sp1 * Sp2
  )

  probs_pop2 <- c(
    pi2 * Se1 * Se2 + (1 - pi2) * (1 - Sp1) * (1 - Sp2),
    pi2 * Se1 * (1 - Se2) + (1 - pi2) * (1 - Sp1) * Sp2,
    pi2 * (1 - Se1) * Se2 + (1 - pi2) * Sp1 * (1 - Sp2),
    pi2 * (1 - Se1) * (1 - Se2) + (1 - pi2) * Sp1 * Sp2
  )

  # Simulate new counts (multinomial)
  n_pop1 <- sum(counts$pop1)
  n_pop2 <- sum(counts$pop2)

  new_counts_pop1 <- rmultinom(1, size = n_pop1, prob = probs_pop1)
  new_counts_pop2 <- rmultinom(1, size = n_pop2, prob = probs_pop2)
```

```
  new_counts <- list(
    pop1 = c(n11 = new_counts_pop1[1], n10 = new_counts_pop1[2], n01 = new_counts_pop1[3], n00 =
new_counts_pop1[4]),
    pop2 = c(n11 = new_counts_pop2[1], n10 = new_counts_pop2[2], n01 = new_counts_pop2[3], n00 =
new_counts_pop2[4])
  )

  # Re-fit model to bootstrap sample (logistic parametrization)
  boot_fit <- optim(
    par = init_logit,
    fn = loglik_logit,
    counts = new_counts,
    method = "BFGS",
    control = list(maxit = 1000)
  )

  # Store bootstrap estimates on probability scale
  boot_estimates[b, ] <- expit(boot_fit$par)
}

# Compute 95% bootstrap percentile CIs
boot_CIs <- apply(boot_estimates, 2, quantile, probs = c(0.025, 0.975))

# Print bootstrap CIs
cat("Bootstrap 95% CIs (probability scale):\n")
param_names <- c("pi1", "pi2", "Se1", "Sp1", "Se2", "Sp2")
for (i in 1:6) {
  cat(param_names[i], ": ", expit(fit_logit$par[i]),
      " (", boot_CIs[1,i], ", ", boot_CIs[2,i], ")\n")
}
```

📚 Summary

- The **bootstrap loop** stays conceptually the same
- The difference is:
  - You use the **logistic-parametrized MLEs** (via `expit()`)
  - You fit using `loglik_logit()`
  - You store bootstrap estimates **on probability scale** (for easy interpretation)

If you like, I can also prepare a **complete final R script** for you that includes:

✅ logistic model
✅ MLE
✅ Wald CIs
✅ Bootstrap CIs

→ all in **one clean file** — ready to run 🚀

Would you like me to generate this complete file for you? Just say the word! 🚀📊

Could you also perform the calculation of the 95% Confidence intervals via the hessian?

Absolutely! Let's go step by step — since you're now using the **logistic parametrization**, here is how to properly compute the **95% CIs from the Hessian**.

🚀 Concept

We are now estimating on the **logit scale**:

$$\theta = (\theta_{\pi_1}, \theta_{\pi_2}, \theta_{Se_1}, \theta_{Sp_1}, \theta_{Se_2}, \theta_{Sp_2})$$

The optimizer **`optim()`** gives:

$$\hat{\theta}$$

You then compute the **Hessian of the negative log-likelihood**, invert it to get the **variance-covariance matrix**:

$$\text{Var}(\hat{\theta}) \approx [H]^{-1}$$

Then, you get **standard errors on the logit scale**:

$$SE(\hat{\theta}_i) = \sqrt{\text{Var}(\hat{\theta}_i)}$$

Finally, you compute **95% Wald CIs on the logit scale**, and transform them back to **probability scale** using the **`expit()`**.

🚀 Full R code

Add this **after your `fit_logit` optimization**:

```r
# Compute Hessian numerically
library(numDeriv)

# Hessian of loglik_logit at MLE
hess_logit <- hessian(func = loglik_logit, x = fit_logit$par, counts = counts)

# Invert Hessian → variance-covariance matrix
vcov_logit <- solve(hess_logit)

# Standard errors on logit scale
se_logit <- sqrt(diag(vcov_logit))

# Wald 95% CI on logit scale
logit_CI_lower <- fit_logit$par - 1.96 * se_logit
logit_CI_upper <- fit_logit$par + 1.96 * se_logit

# Transform to probability scale
expit_CI_lower <- expit(logit_CI_lower)
expit_CI_upper <- expit(logit_CI_upper)

# Print estimates with 95% Wald CIs (probability scale)
cat("Wald 95% CIs (probability scale):\n")
param_names <- c("pi1", "pi2", "Se1", "Sp1", "Se2", "Sp2")
for (i in 1:6) {
  cat(param_names[i], ": ", expit(fit_logit$par[i]),
      " (", expit_CI_lower[i], ", ", expit_CI_upper[i], ")\n")
}
```

🚀 Summary of steps

1️⃣ Compute **Hessian** at MLE
2️⃣ Invert Hessian → **variance-covariance matrix**
3️⃣ Get **standard errors**
4️⃣ Compute **CIs on logit scale**
5️⃣ **Transform back to probability scale** with `expit()`
6️⃣ Print **final CIs**

✅ Advantages

- This is the **correct way** to compute CIs when using logistic parametrization
- Works perfectly with your current `loglik_logit()` model
- No need for bounds anymore — smooth optimization

🚀 Final note

The Wald CIs are usually **fast** but can be a bit approximate.
The **bootstrap CIs** are more robust — especially in small samples or near boundary values (Se/Sp near 1).

👉 I recommend you compute **both** — and compare. Often they are very close — sometimes bootstrap is wider (more conservative).

Would you like me to generate a **final full R script** that combines:

✅ Logistic model
✅ MLE
✅ Hessian-based CIs
✅ Bootstrap CIs

... so you have **one complete, ready-to-use script**?

Just say **yes** — I will generate it for you! 🚀📊

Please provide a comprehensive R code for the issues discussed in this session.

Perfect — here is a **comprehensive and clean R script** that implements *everything* we discussed:

✅ Hui-Walter model
✅ Logistic parametrization
✅ MLE estimation (`optim`)
✅ Hessian-based (Wald) 95% CIs
✅ Parametric Bootstrap 95% CIs

🚀 Full R script

```r
# Hui-Walter model with logistic parametrization, Wald CIs, and Bootstrap CIs
# Comprehensive example

#-------------------------------
# 1. Setup: Observed counts
#-------------------------------

# Observed counts from example
counts <- list(
  pop1 = c(n11 = 50, n10 = 20, n01 = 15, n00 = 100),
  pop2 = c(n11 = 30, n10 = 10, n01 = 25, n00 = 200)
)

#-------------------------------
# 2. Logistic (expit) function
#-------------------------------
expit <- function(x) {
  1 / (1 + exp(-x))
}

#-------------------------------
# 3. Log-likelihood function (logistic parametrization)
#-------------------------------
loglik_logit <- function(par, counts) {
  # Transform parameters to probability scale
  pi1 <- expit(par[1])
  pi2 <- expit(par[2])
  Se1 <- expit(par[3])
  Sp1 <- expit(par[4])
  Se2 <- expit(par[5])
  Sp2 <- expit(par[6])

  # Probabilities for each test outcome in each population
  # Population 1
  p11_1 <- pi1 * Se1 * Se2 + (1 - pi1) * (1 - Sp1) * (1 - Sp2)
  p10_1 <- pi1 * Se1 * (1 - Se2) + (1 - pi1) * (1 - Sp1) * Sp2
  p01_1 <- pi1 * (1 - Se1) * Se2 + (1 - pi1) * Sp1 * (1 - Sp2)
  p00_1 <- pi1 * (1 - Se1) * (1 - Se2) + (1 - pi1) * Sp1 * Sp2

  # Population 2
  p11_2 <- pi2 * Se1 * Se2 + (1 - pi2) * (1 - Sp1) * (1 - Sp2)
  p10_2 <- pi2 * Se1 * (1 - Se2) + (1 - pi2) * (1 - Sp1) * Sp2
  p01_2 <- pi2 * (1 - Se1) * Se2 + (1 - pi2) * Sp1 * (1 - Sp2)
  p00_2 <- pi2 * (1 - Se1) * (1 - Se2) + (1 - pi2) * Sp1 * Sp2

  # Add small constant to avoid log(0)
  eps <- 1e-10

  # Log-likelihood
  ll <- 0
  ll <- ll + counts$pop1["n11"] * log(p11_1 + eps)
  ll <- ll + counts$pop1["n10"] * log(p10_1 + eps)
  ll <- ll + counts$pop1["n01"] * log(p01_1 + eps)
  ll <- ll + counts$pop1["n00"] * log(p00_1 + eps)

  ll <- ll + counts$pop2["n11"] * log(p11_2 + eps)
  ll <- ll + counts$pop2["n10"] * log(p10_2 + eps)
  ll <- ll + counts$pop2["n01"] * log(p01_2 + eps)
  ll <- ll + counts$pop2["n00"] * log(p00_2 + eps)

  return(-ll)  # because optim does minimization
}

#-------------------------------
# 4. Initial values (logit scale)
#-------------------------------
# logit(0.5) = 0
# logit(0.8) ≈ 1.386
# logit(0.9) ≈ 2.197

init_logit <- c(0, 0, 1.386, 2.197, 1.386, 2.197)
```

```r
#-------------------------------
# 5. MLE estimation with optim
#-------------------------------
fit_logit <- optim(
  par = init_logit,
  fn = loglik_logit,
  counts = counts,
  method = "BFGS",
  control = list(trace = 1, maxit = 1000)
)

# Extract MLE estimates on probability scale
param_names <- c("pi1", "pi2", "Se1", "Sp1", "Se2", "Sp2")
mle_estimates <- expit(fit_logit$par)

cat("\nEstimated parameters (MLE, probability scale):\n")
for (i in 1:6) {
  cat(param_names[i], ": ", mle_estimates[i], "\n")
}

#-------------------------------
# 6. Hessian-based Wald 95% CIs
#-------------------------------
library(numDeriv)

# Compute Hessian
hess_logit <- hessian(func = loglik_logit, x = fit_logit$par, counts = counts)

# Invert Hessian to get variance-covariance matrix
vcov_logit <- solve(hess_logit)

# Standard errors on logit scale
se_logit <- sqrt(diag(vcov_logit))

# Wald 95% CI on logit scale
logit_CI_lower <- fit_logit$par - 1.96 * se_logit
logit_CI_upper <- fit_logit$par + 1.96 * se_logit

# Transform to probability scale
expit_CI_lower <- expit(logit_CI_lower)
expit_CI_upper <- expit(logit_CI_upper)

cat("\nWald 95% CIs (probability scale):\n")
for (i in 1:6) {
  cat(param_names[i], ": ", mle_estimates[i],
      " (", expit_CI_lower[i], ", ", expit_CI_upper[i], ")\n")
}

#-------------------------------
# 7. Parametric Bootstrap 95% CIs
#-------------------------------
n_boot <- 1000
boot_estimates <- matrix(NA, nrow = n_boot, ncol = 6)

set.seed(123)  # for reproducibility

for (b in 1:n_boot) {
  # Use MLE estimates to simulate new data
  pi1 <- mle_estimates[1]
  pi2 <- mle_estimates[2]
  Se1 <- mle_estimates[3]
  Sp1 <- mle_estimates[4]
  Se2 <- mle_estimates[5]
  Sp2 <- mle_estimates[6]

  # Probabilities of each cell
  probs_pop1 <- c(
    pi1 * Se1 * Se2 + (1 - pi1) * (1 - Sp1) * (1 - Sp2),
    pi1 * Se1 * (1 - Se2) + (1 - pi1) * (1 - Sp1) * Sp2,
    pi1 * (1 - Se1) * Se2 + (1 - pi1) * Sp1 * (1 - Sp2),
    pi1 * (1 - Se1) * (1 - Se2) + (1 - pi1) * Sp1 * Sp2
  )

  probs_pop2 <- c(
    pi2 * Se1 * Se2 + (1 - pi2) * (1 - Sp1) * (1 - Sp2),
```

```
      pi2 * Se1 * (1 - Se2) + (1 - pi2) * (1 - Sp1) * Sp2,
      pi2 * (1 - Se1) * Se2 + (1 - pi2) * Sp1 * (1 - Sp2),
      pi2 * (1 - Se1) * (1 - Se2) + (1 - pi2) * Sp1 * Sp2
    )

    # Simulate new counts
    n_pop1 <- sum(counts$pop1)
    n_pop2 <- sum(counts$pop2)

    new_counts_pop1 <- rmultinom(1, size = n_pop1, prob = probs_pop1)
    new_counts_pop2 <- rmultinom(1, size = n_pop2, prob = probs_pop2)

    new_counts <- list(
      pop1 = c(n11 = new_counts_pop1[1], n10 = new_counts_pop1[2], n01 = new_counts_pop1[3], n00 =
  new_counts_pop1[4]),
      pop2 = c(n11 = new_counts_pop2[1], n10 = new_counts_pop2[2], n01 = new_counts_pop2[3], n00 =
  new_counts_pop2[4])
    )

    # Re-fit model to bootstrap sample
    boot_fit <- optim(
      par = init_logit,
      fn = loglik_logit,
      counts = new_counts,
      method = "BFGS",
      control = list(maxit = 1000)
    )

    # Store bootstrap estimates on probability scale
    boot_estimates[b, ] <- expit(boot_fit$par)
  }

  # Compute bootstrap percentile CIs
  boot_CIs <- apply(boot_estimates, 2, quantile, probs = c(0.025, 0.975))

  cat("\nBootstrap 95% CIs (probability scale):\n")
  for (i in 1:6) {
    cat(param_names[i], ": ", mle_estimates[i],
        " (", boot_CIs[1,i], ", ", boot_CIs[2,i], ")\n")
  }
```

🚀 What this full script does

✅ Defines the Hui-Walter **latent class model** with logistic parametrization
✅ Estimates **MLE** with `optim()`
✅ Computes **Hessian-based 95% Wald CIs**
✅ Performs **parametric bootstrap** and computes **bootstrap 95% CIs**
✅ Outputs **all results** — clearly labeled and easy to read

🚀 Next possible extensions

✅ Add **profile likelihood** CIs (optional)
✅ Add **Bayesian model in Stan** (super clean with logistic parametrization)
✅ Add **simulation study** (generate synthetic data and test recovery of parameters)

Would you like me to also:

✅ Generate the **Bayesian Stan version** for this model?
✅ Generate a **simulation study example**?
✅ Generate a version with **more than 2 populations**?

Just tell me — I’m happy to help! 🚀📊
